# Impacts of elevated atmospheric CO_2_ on nutrient content of important food crops

**DOI:** 10.1038/sdata.2015.36

**Published:** 2015-07-21

**Authors:** Lee H. Dietterich, Antonella Zanobetti, Itai Kloog, Peter Huybers, Andrew D. B. Leakey, Arnold J. Bloom, Eli Carlisle, Nimesha Fernando, Glenn Fitzgerald, Toshihiro Hasegawa, N. Michele Holbrook, Randall L. Nelson, Robert Norton, Michael J. Ottman, Victor Raboy, Hidemitsu Sakai, Karla A. Sartor, Joel Schwartz, Saman Seneweera, Yasuhiro Usui, Satoshi Yoshinaga, Samuel S. Myers

**Affiliations:** 1 Department of Biology, University of Pennsylvania, Philadelphia, Pennsylvania 19104, USA; 2 Department of Environmental Health, Harvard T. H. Chan School of Public Health, Boston, Massachusetts 02215, USA; 3 The Department of Geography and Environmental Development, Ben-Gurion University of the Negev, PO Box 653, Beer Sheva, Israel; 4 Department of Earth and Planetary Science, Harvard University, Cambridge, Massachusetts 02138, USA; 5 Department of Plant Biology and Institute for Genomic Biology, University of Illinois at Urbana-Champaign, Urbana, Illinois 61801, USA; 6 Department of Plant Sciences, University of California at Davis, Davis, California 95616, USA; 7 Department of Land, Air, & Water Resources, University of California at Davis, Davis, California 95616, USA; 8 Faculty of Veterinary and Agricultural Sciences, The University of Melbourne, Victoria 3010, Australia; 9 Faculty of Science and Technology, Centre for Environmental Management, Federation University, University Drive, Mount Helen, Victoria 3350, Australia; 10 Department of Economic Development, Jobs, Transport and Resources, Horsham, Victoria 3001, Australia; 11 Agro-Meteorology Division, National Institute for Agro-Environmental Sciences, Tsukuba, Ibaraki 305-8604, Japan; 12 Department of Organismic and Evolutionary Biology, Harvard University, Cambridge, Massachusetts 02138, USA; 13 United States Department of Agriculture, Agricultural Research Service, Soybean/Maize Germplasm, Pathology, and Genetics Research Unit, Department of Crop Sciences, University of Illinois, Urbana, Illinois 61801, USA; 14 International Plant Nutrition Institute, 54 Florence St, Horsham, Victoria 3400, Australia; 15 School of Plant Sciences, University of Arizona, Tucson, Arizona 85721, USA; 16 United States Department of Agriculture Agricultural Research Service, Aberdeen, Idaho, 83210, USA; 17 Compa Industries, Inc., Los Alamos National Laboratory Environmental Protection Division, Los Alamos, New Mexico 87545, USA; 18 Center for Crop Health, University of Southern Queensland, Toowoomba, QLD 4350, Australia; 19 Hokuriku Research Center, NARO Agricultural Research Center, National Agricultural and Food Research Organization, Joetsu, Niigata 943-0193, Japan; 20 Harvard University Center for the Environment, Cambridge, Massachusetts 02138, USA

**Keywords:** Environmental health, Risk factors, Plant physiology, Plant breeding

## Abstract

One of the many ways that climate change may affect human health is by altering the nutrient content of food crops. However, previous attempts to study the effects of increased atmospheric CO_2_ on crop nutrition have been limited by small sample sizes and/or artificial growing conditions. Here we present data from a meta-analysis of the nutritional contents of the edible portions of 41 cultivars of six major crop species grown using free-air CO_2_ enrichment (FACE) technology to expose crops to ambient and elevated CO_2_ concentrations in otherwise normal field cultivation conditions. This data, collected across three continents, represents over ten times more data on the nutrient content of crops grown in FACE experiments than was previously available. We expect it to be deeply useful to future studies, such as efforts to understand the impacts of elevated atmospheric CO_2_ on crop macro- and micronutrient concentrations, or attempts to alleviate harmful effects of these changes for the billions of people who depend on these crops for essential nutrients.

## Background & Summary

Climate change may have numerous effects on human health, not least via effects on agriculture and nutrition. Because plant productivity is fundamentally tied to atmospheric CO_2_ by photosynthesis, changes in atmospheric CO_2_ concentration ([CO_2_]) may have cascading effects on numerous aspects of plant biochemistry. If these cascading effects include changes in the nutrient content of staple crops, this could have substantial implications for public health in regions where people rely on those crops for critical nutrients. Indeed, nutrient deficiencies are already a major global public health problem^1–3^.

Previous attempts to examine how increasing atmospheric [CO_2_] affects crop nutrition have been hampered by artificial growing conditions and/or small sample sizes. For instance, there were several reports of decreases in zinc, iron, and protein in wheat^4–7^, barley^5^, and rice^8^ grown in outdoor open-topped chambers or in indoor climate-controlled growth chambers. However, the advent of free-air CO_2_ enrichment (FACE) technology has allowed researchers to grow plants under standard field management practices while precisely manipulating local CO_2_ concentrations. Lieffering *et al.*^9^ grew rice in a FACE experiment more representative of standard field conditions and found no effect of elevated [CO_2_] on nutrients other than nitrogen. These authors suggested that previous significant results may be due to unintentional nutrient limitation caused by growing plants in pots. Several subsequent FACE experiments did find decreased nutrient concentrations associated with elevated [CO_2_] in soybean^10^, sorghum^10^, potatoes^11^, wheat^12–14^, and barley^15^, but many of these studies lacked the sample sizes necessary to determine whether potentially meaningful differences were significant.

Myers *et al.*^16^ grew and analyzed samples of rice (*Oryza sativa*, 18 cultivars), wheat (*Triticum aestivum*, 8 cultivars), maize (*Zea mays*, 2 cultivars), soybeans (*Glycine max*, 7 cultivars), field peas (*Pisum sativum*, 5 cultivars), and sorghum (*Sorghum bicolor*, 1 cultivar) under ambient versus elevated [CO_2_] in FACE experiments between 1998–2010. Ambient [CO_2_] was between 364–386 ppm and elevated [CO_2_] was between 546–586 ppm across all study sites, consistent with the lower end of global [CO_2_] predictions for the next 40–60 years^17^. We reported nutrient concentrations of the edible portions of these crops, and found that elevated [CO_2_] decreased many of their zinc, iron, and sometimes protein concentrations^16^.

These effects varied among plant functional types, with C_3_ grasses and legumes consistently affected and C_4_ plants less so. Effects on nutrient concentrations varied among different cultivars of the same crop species. Both large-scale (e.g., C_3_/C_4_ photosynthetic pathways) and small-scale (intraspecific) differences in plant physiology contribute to the effects observed. Interestingly, effects of elevated [CO_2_] also differed among 12 nutrients measured, indicating mechanisms more complex than carbohydrate dilution^18,19^. This study improved on previous work by using more crops, larger sample sizes, and FACE methods more representative of field conditions than older techniques such as open-topped chambers or fully enclosed growth chambers, resulting in over tenfold more samples than were previously available^16^, though see Loladze^20^.

Here we report the raw data presented by Myers *et al.*^16^ for use in further studies on the role of atmospheric [CO_2_] and other cultivation conditions on the nutrient content of food crops. While some aggregated data have been presented concerning agronomic response to elevated [CO_2_]^13,14,21,22^, we report cultivation conditions including water availability, fertilizer application, temperature, and growing season length, along with the nutrient concentrations of edible portions of crops. We also report edible tissue concentrations of phytic acid, a compound that has been shown to inhibit absorption of zinc and other nutrients in mammalian guts, and is mathematically related to bioavailable zinc uptake through the Miller equation^23^.

These data are expected to permit detailed analysis of the roles of cultivation conditions, including [CO_2_], on crop plants’ nutritional content, as well as more basic studies of their biochemistry that may include numerous nutrients simultaneously. This information will help identify threats to the health of billions of people worldwide who rely on these crops for essential nutrients. It will also provide insights about how to address these threats, for instance, by revealing which cultivars are more likely to provide more nutrition under elevated [CO_2_], thereby informing future crop breeding efforts.

## Methods

### Cultivation

Crops were grown in FACE experiments with paired designs, such that samples grown at ambient [CO_2_] (approximately 380 ppm) could be compared with samples grown under identical conditions except that [CO_2_] was elevated to approximately 550 ppm during daylight hours (during both day and night in the case of sorghum, and elevated to approximately 580 ppm, or 200 ppm above ambient, in the case of rice). More recent work has suggested that elevating [CO_2_] 24 h d^−1^ may give a more complete picture of the effects of climate change on plant physiology and biochemistry^24^. Many of these experiments also tested other variables, such as cultivar, irrigation, nitrogen application, and temperature. Detailed agronomic methods are available for wheat^25^, soybean^26^, maize^27^, sorghum^28^, rice^29^, and field peas^25^. The following descriptions outline the experimental designs for each crop, which underlie the structure of the data we present.

### Wheat

Wheat samples (*Triticum aestivum*) were grown in Horsham, Victoria, Australia between 2007–2009. In 2007 and 2008, cultivars Yitpi and Janz were grown under full factorial manipulations of daytime [CO_2_] (ambient or elevated), irrigation (rainfed only or supplemental irrigation), and time of sowing (early or late), with four replicates per cultivar per year. Crops sown early, consistent with local practice, experience a cool, wet early season and a warmer, drier, late season. Late time of sowing was used to simulate predicted climate change more holistically by moving the growing season toward warmer and drier conditions. For cultivar Yitpi, these treatments were crossed further with a nitrogen (N) fertilizer treatment, with some plants receiving N fertilizer and others receiving none; cultivar Janz received no N fertilizer. Superphosphate was drilled with the seed at sowing at 7–9 kg P ha^−1^ and 8–11 kg S ha^−1^ each year, according to local practice. In 2009, this experiment was continued for cultivars Yitpi and Janz, and six more cultivars (Drysdale, Gladius, H45, Hartog, Silverstar, and Zebu) were added and exposed to the same treatments as Janz, again with four replicates per cultivar.

Additional samples of cultivar Yitpi were grown in Walpeup, Victoria, Australia (details published^21^) in 2008 and 2009. Time of sowing (early or late) and daytime [CO_2_] (ambient or elevated) were crossed in a full factorial design with four replicates per treatment per year. All plants received some supplemental irrigation in one season to avoid total crop loss to drought, and none received N fertilizer, as pre-sowing soil nitrate concentrations were high. Superphosphate was drilled at sowing at rates of 9 kg P ha^−1^ and 11 kg S ha^−1^ each year. Rainfall, temperature, and other meteorological conditions were recorded daily using automatic weather stations. Several papers analyzing these data have been published^13,14,21,22^.

### Soybean

Soybean (*Glycine max*) was grown in Champaign, Illinois, USA between 2001–2008. The only growth condition manipulated was daytime [CO_2_] (ambient or elevated); plants received neither irrigation nor fertilizer. The choice of cultivars varied among years. In 2001, 2002, and 2004, seven cultivars were planted: Clark, Dwight, Flyer, Loda, Pana, Spencer, and Williams. In 2006 and 2007, only cultivar Pana was planted, and in 2008, only cultivars Pana and Loda were planted. In every case, samples were grown and collected from four replicates per cultivar per year.

In most cases, precipitation was recorded for the entire growing season. For the 2002 soybean crop, precipitation data were available for May 15 to September 30, but the growing season was June 1 to October 16. The presented precipitation value is total precipitation from June 1 to September 30; it may be more accurate to add the additional 40.5 mm of precipitation from May 15–31 to compensate for the lack of precipitation data for October 1–16.

### Maize

Maize (*Zea mays*) was grown in Champaign, Illinois, USA in 2008. The two commercial hybrids Cv34B43 (Pioneer Hi-Bred International) and DKC61–19 (Dekalb) were both subjected to full factorial combinations of daytime [CO_2_] (ambient or elevated) and nitrogen fertilizer (present or absent), with four replicates per treatment per hybrid. Maize did not receive any irrigation. Although the hybrids used are not cultivars in the technical sense, we refer to them as cultivars elsewhere in this paper and dataset for simplicity.

### Sorghum

Sorghum (*Sorghum bicolor*) was grown in Maricopa, Arizona, USA in 1998 and 1999. Atmospheric [CO_2_] (ambient or elevated 24 h d^−1^) and irrigation (low or high) treatments were varied in a full factorial design with four replicates per treatment per year. All crops received roughly equal applications of nitrogen and phosphorus fertilizer in both years.

### Rice

Rice (*Oryza sativa*) was grown in Shizukuishi, Iwate, Japan in 2007 and 2008, and in Tsukubamirai, Ibaraki, Japan, in 2010. Rice fields in both sites were flooded for most of the growing season consistent with local practices. In 2007, three replicates of cultivar Akitakomachi were grown in Shizukuishi in a full factorial experiment crossing daytime [CO_2_] (ambient or elevated by approximately 200 ppm^30^) and temperature (normal, or elevated 2 °C by heating cables installed on the submerged soil surface^31^). In 2008, the 2007 experiment was repeated, and three replicates each of cultivars Koshihikari and Takanari were grown under ambient or elevated daytime [CO_2_] at normal (unmodified) temperatures. All rice grown in Shizukuishi received NPK fertilizer consistent with local practices.

A more complex rice experiment was grown in Tsukubamirai in 2010. Experimental variables included daytime [CO_2_] (ambient or elevated by approximately 200 ppm^32^), nitrogen application (none, standard, or increased 50–100% above standard levels), and temperature (ambient or elevated 2 °C by heating cables installed on the submerged soil surface^31^). There were four replicates per cultivar per treatment combination, but while daytime [CO_2_] was varied within each treatment, the rest of the design was not fully factorial. For 14 cultivars, only daytime [CO_2_] was varied and temperature was not manipulated. Of these, cultivars Aikoku, Akidawara, Akihikari, Akita63, Akitakomachi, and Norin8 received standard N application and cultivars Bekoaoba, Hoshiaoba, IR72, Hokuriku193, Lemont, Milyang23, Momiroman, and Nipponbare received increased N application. Cultivar Takanari was grown at ambient temperature under fully crossed daytime [CO_2_] and nitrogen (standard and increased) treatments. Cultivar SY63 was grown with standard N applications under fully crossed daytime [CO_2_] and temperature (ambient or increased) treatments. For cultivars 86Y8 and Koshihikari, all three variables (daytime [CO_2_], nitrogen, and temperature) were manipulated. Cultivar 86Y8 received only standard and increased N applications, while Koshihikari received zero, standard, and increased N applications. Samples of these two cultivars receiving standard N application were exposed to both ambient and increased temperature treatments, with other N treatments being grown only at ambient temperature. Increased N application levels were 100% above standard for all cultivars except Koshihikari, for which increased N applications were 50% above standard levels. All rice grown in Tsukubamirai received phosphorus and potassium fertilizer consistent with local practices.

### Field peas

Field peas were grown in Horsham, Victoria, Australia in a full factorial experiment crossing daytime [CO_2_] (ambient or elevated) with supplemental irrigation (present or absent). Eight replicates of each of five cultivars (Bohatyr, Kaspa, OZP0601, OZP0902, and Sturt) were grown in 2010. Plants did not receive nitrogen fertilizer, but received superphosphate at rates of 9 kg P ha^−1^ and 11 kg S ha^−1^.

### Elemental analysis

Samples of the edible portions of each crop were dried and ground for elemental analysis. Nitrogen concentration of soybeans, maize, sorghum, rice, and peas was determined by flash combustion coupled with thermal conductivity/IR detection of the resulting N_2_, NO_x_, and CO_2_ gases on a LECO TruSpec CN Analyzer^33^. Nitrogen concentration of wheat was measured by a similar technique on a LECO TruMac calibrated with EDTA^34^.

Inductively coupled plasma optical emission spectroscopy (ICP-OES) was used to measure sample P, K, S, B, Ca, Mg, Zn, Mn, Fe, and Cu concentrations. Wheat samples were prepared by sample digestion in nitric acid^35^. Samples of all other crops were digested by microwave heating in closed vessels with nitric acid and hydrogen peroxide. All nutrient concentrations are reported at 0% grain moisture concentration.

### Phytic acid analysis

Phytic acid concentrations in the edible portions of crops were determined using a modification of the method of Haug and Lantzsch (1983; hereafter referred to as the ‘HL’ method)^36^. This method takes advantage of the ability of phytic acid to precipitate iron from a solution and consists of two basic steps. First, extracts containing phytic acid are incubated with solutions with known iron concentration. Following precipitation of iron phytate, the iron remaining in solution is quantitated. To determine phytic acid in tissue extracts, the results are compared with those obtained using a phytic acid standard curve. We express phytic acid as its phosphorus content (phytic acid phosphorus). One can convert phytic acid phosphorus to phytic acid by multiplying by 3.548.

**Figure 1:**
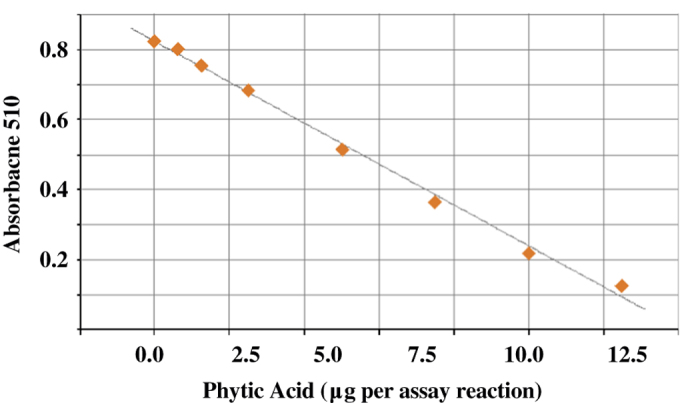
A typical standard curve used to assay phytic acid using the HL method. The known concentrations of phytic acid standards are plotted against their absorbance at 510 nm after sample processing. Please see text for details.

Tissue samples were stored in a desiccator until analysis. If the samples received were whole grains, they were first milled to pass 40 mesh. For the iron precipitation step, 50 mg of tissue were extracted in 1.0 ml of extraction media (0.2 M HCL:10% Na_2_SO_4_) in 1.5 ml Eppendorf tubes overnight at 4 °C with shaking. Extracts were centrifuged (4,500 RCF, 20 min). The iron precipitations were set up as follows. A 25 μl aliquot of extract was placed in a second Eppendorf tube, to which was added 225 μl of extraction media and 500 μl of iron solution (0.2 gm Ammonium iron (III) sulfate dodecahydrate dissolved in 1.0 L 0.2 N HCl). A phytic acid phosphorus standard curve was set up similarly except that instead of aliquots of extract, 25 μl aliquots of phytic acid standards (see below) were used, to give from 0.625 to 12.5 μg phytic acid P per assay (Fig. 1). All tubes were then placed in a boiling water bath for 30 min, cooled to room temperature, and centrifuged (4,500 RCF:20 min). Aliquots (100 μl) of precipitation supernatant were pipetted into microtitre plate wells and 150 μl of ‘HL reagent’ (5.0 g 2,2'-bipyridine and 5.0 ml thioglycolic acid dissolved in 500 ml distilled water) was added. The plates were then read on a microtitre plate spectrophotometer at 510 nm. Duplicate extractions were conducted for each tissue sample and for each extraction, duplicate precipitations were conducted. The colorimetric assay of precipitation supernatant was conducted in triplicate. If the mean values obtained for the two extractions of a given tissue sample were not within 15% agreement, a third extraction was conducted, and the two values in best agreement were used to calculate sample mean.

The phytic acid standard curve was prepared using commercially-obtained phytic acid (Sigma): a phytic acid dodecasodium salt hydrate. A stock solution of 100 ml of 1.0 mg phytic acid P ml^−1^ was prepared as follows: 549.9 mg Sigma phytic acid dodecasodium salt hydrate was dissolved into either 100 ml of 0.8 N HCl or 100 ml of 0.2 N HCl [(1.0 mg PAP/ml)(100 ml)(1022.8 g phytic acid dodecasodium salt hydrate/186 g PAP)=549.9 mg phytic acid dodecasodium salt hydrate per 100 ml]. These 1.0 mg ml^−1^ stocks were then used to prepare standard solutions that are 25, 50, 100, 200, 300, 400, and 500 μg phytic acid P ml^−1^.

## Data Records

The dataset (Data Citation 1) is structured such that each row contains all of our data from a single plant sample, with columns representing growing conditions and resulting nutrient concentrations. Cells marked ‘NA’ indicate missing, unavailable, or inapplicable data. Column variable definitions are provided in Table 1 (available online only).

## Technical Validation

### Elemental analysis

Element concentration measurements were validated in two ways. First, experimental samples were interspersed with standard reference materials of known element concentrations, whose measured concentrations were confirmed to be within their certified acceptable ranges. Second, for most crops, every tenth sample was run in duplicate to ensure consistency; fewer than 5% of element concentration measurements in duplicated samples differed by more than 10%. For wheat samples, every 25th sample was run in duplicate. All results were different by less than 10%, and the series was reanalyzed if duplicates differed by more than 5%.

### Phytic acid analysis

The following procedures were used to test the accuracy and reproducibility of the phytic acid assay method used here.

We first verified the concentration of the commercially-obtained phytic acid (Sigma) used to prepare the phytic acid standard curve. This reagent is a phytic acid dodecasodium salt hydrate, with a dodecasodium salt formula weight of 923.8, and a water content of 5.5 moles H_2_O per mole phytic acid. This product would then be 90.3% phytic acid dodecasodium salt with a phytic acid dodecasodium•H_2_O formula weight of 1022.8. To confirm this, two standard solutions were prepared with this product and were assayed six times, using the ‘ferric precipitation’ assay described below, and found to be within 2% of the predicted value, based on the above assumptions. Care was also taken to determine where the phytic acid standard curves produced from this reagent are linear for each method (Fig. 1).

Eight tissue standards with greatly varying levels of endogenous phytic acid were used as controls to indicate accuracy and reliability of the methods used (Table 2). One set of these eight tissue standards were tested for every 100 samples tested. Flours were produced using seed produced by three barley (*Hordeum vulgare* L.) lines^37^, two maize (*Zea mays* L.) hybrids^38^, and three soybean [*Glycine max* L. (Merr.)] lines^39,40^. Seed of the barley lines were produced by the USDA-ARS at Aberdeen, Idaho in 2006. Seed of the maize lines were produced for the USDA-ARS at a winter nursery station near Santiago, Chile in the winter of 2001. Seed of the soybean line CX183 was kindly provided by Seedomics Hybrid Corn Inc. (Forest, IN) and were produced in Indiana in the summer of 2002. Seed of the soybean lines Jack and CAPPA were produced at the University of Missouri, Columbia, in 2008.

The lines included as tissue standards in this study either produced seed with phytic acid levels typical of crops produced today or with reduced seed phytic acid due to either *low phytic acid* (*lpa*) genotype or genetic engineering to over-express the enzyme phytase in seeds. The ‘normal phytic acid’ lines were the barley cv. Harrington (the genetic background in which the *lpa* genotypes included in this study were isolated), a maize hybrid produced by crossing the public inbred lines A632 and A619 (the ‘near-isogenic’ hybrid for the A631XA619 *lpa*1-1 included in this study), and the soybean cultivar ‘Jack’ (the control for the *lpa* CX183 and the ‘high phytase’ ‘CAPPA’, respectively). The ‘reduced phytic acid’ lines were the genotypes barley *lpa*1-1 and barley *lpa*-M955, each in the Harrington background, maize *lpa*1-1 as a A632XA619 isohybrid, soybean CX183, and the genetically-engineered soybean line ‘CAPPA’, in which a bacterial phytase is overexpressed in developing seed.

To calibrate the level of phytic acid in seed produced by these lines, these tissue standards were analyzed for phytic acid phosphorus using two methods previously shown to be highly accurate and reproducible; the ‘ferric precipitation’ method^38^ and a high pressure liquid chromatography (HPLC) method (analyses kindly provided by Dr Sören Rasmussen, University of Copenhagen, Denmark).

The ferric precipitation method assays total, acid-soluble inositol phosphates, referred to here as phytic acid P^38,41^. Previous HPLC analyses indicate that in most genotypes >90% of total seed inositol phosphate consists of phytic acid (inositol hexakisphosphate), with the remainder consisting of less highly phosphorylated inositol phosphates such as inositol tetrakisphosphate or pentakisphosphate^37^. Thus use of the term ‘phytic acid P’ for total inositol phosphate is accurate in most cases. Aliquots of tissue (0.5 gm) were extracted in 0.4 M HCl:0.7 M Na_2_SO_4_ by shaking overnight at 4 °C. Following centrifugation (10,000 *g* for 10 min) and filtering through Whatman #1, phytic acid P in 10 ml of supernatant was obtained as a ferric precipitate, wet-ashed and assayed for P colorimetrically. Phytic acid P is expressed in terms of its P (atomic weight 31) content to facilitate comparisons between seed P fractions and methods of determination. All assays were conducted in triplicate.

For determination of flour phytic acid P using HPLC, flour samples (40 mg) were extracted in 1.6 ml 0.5 M HCl (30% Suprapur, Merck) by shaking overnight at 4 °C. Following centrifugation (10,000 *g* for 10 min) supernatants were filtered through a 0.45 μm syringe filter (Pall Life Sciences). Aliquots of filtered extract (100 μl) were fractionated via ion-exchange chromatography using a chemically inert HPLC system 10Avp series (Shimadzu, Kyoto, Japan) equipped with a CarboPac PA-100 column (Dionex). Phytic acid peaks were detected post-column as described by Bohn *et al.*^42^ following the protocol of Carlsson *et al.*^43^, and quantitated via comparison with a standard curve prepared using reagent grade phytic acid (Sigma). The range of quantification was 1 to 30 nmol phytic acid per 100 μl sample injected. All assays were conducted in triplicate.

The accuracy and reproducibility of the ‘HL’ method used here is illustrated by comparing the means and Standard Deviation of the Means (‘Standard Errors’) obtained in an HL method run with that obtained with these other two methods (Table 2).

## Additional Information

Table 1 is only available in the online version of this paper.

**How to cite this article:** Dietterich, L. H. *et al.* Impacts of elevated atmospheric CO_2_ on nutrient content of important food crops. *Sci. Data* 2:150036 doi: 10.1038/sdata.2015.36 (2015).

## Supplementary Material



## Figures and Tables

**Table 1:** Definitions of reported variables, corresponding to columns of the dataset (Data Citation 1)

**Variable**	**Definition**
Row	An index, numbered 2–1153, to aid in sorting the dataset.
Study	Name of the study in which the plants were grown.
Data_Type	Indicates whether samples are raw data for use in analyses (‘RawData’), or duplicate samples used for technical validation (‘Validation’).
City	City in which samples were grown.
State	State in which samples were grown.
Country	Country in which samples were grown.
Year	Year in which samples were grown.
Crop	Crop species grown. Either wheat (*Triticum aestivum*), soybean (*Glycine max*), sorghum (*Sorghum bicolor*), corn (*Zea mays*), rice (*Oryza sativa*), or field peas (‘peas,’ *Pisum sativum*).
Elevated_CO2_Target_ppm	Target concentration for elevated [CO_2_] treatments, in parts per million (ppm).
Elevated_CO2_Achieved_ppm	Daytime average [CO_2_] in elevated [CO_2_] treatments, in ppm.
Ambient_CO2_ppm	Daytime average [CO_2_] in ambient (control) [CO_2_] treatments, in ppm.
FACE_Ring_Diameter_m	Diameter of FACE rings, in meters.
SampleCode	A unique number or character string identifying each sample.
Replicate	Denotes the experimental replicate, sometimes called ‘block’ in the accompanying literature, in which samples were grown.
Ring	Denotes the specific FACE ring in which samples were grown.
Plot	Location within a FACE ring in which samples were grown. This may differ among paired samples within a replicate.
Temperature_Qualitative	Temperature at which crops were grown; either ‘Ambient,’ for most crops, or ‘Elevated’ by 2 °C for some rice samples.
Water_Qualitative	Studies used up to two irrigation treatments. For studies using two irrigation treatments, ‘Wet’ and ‘Dry’ refer to samples that received more and less water, respectively. For studies that did not vary irrigation, this is ‘NA.’
Precipitation_mm	Water crops received as precipitation during the growing season, in mm.
Irrigation_mm	Water crops received as irrigation during the growing season, in mm.
Total_Water_mm	Sum of precipitation and irrigation; the total amount of water available to crops during the growing season, in mm.
Nitrogen_Application_Qualitative	Studies used up to three levels of nitrogen fertilizer application per cultivar. ‘Low’ means no nitrogen applied to crops, ‘Medium’ means some nitrogen fertilizer applied to crops, and ‘High’ is reserved for greater amounts of nitrogen application than ‘Medium’ samples. ‘High’ nitrogen applications vary among rice cultivars.
Nitrogen_Application_Quantitative	Amount of nitrogen applied to crops as fertilizer, in kg N ha^−1^.
Phosphorus_Application_Quantitative	Amount of phosphorus applied to crops as fertilizer, in kg P ha^−1^.
Potassium_Application_Quantitative	Amount of potassium applied to crops as fertilizer, in kg K ha^−1^.
Sowing_Timing_Qualititative	Time of sowing (TOS) was an independent variable for the wheat experiments. ‘TOS1’ represents late autumn planting, typical for the region, and ‘TOS2’ represents a mid-winter planting. In general, ‘TOS2’ crops matured under hotter, drier conditions. For all other crops this is ‘NA.’
Sowing_Timing_Quantitative	Date that crops were sown (mm/dd/yy). For rice, we present the date that seedlings were transplanted into hills in the field; seeds were planted in seedling trays approximately one month prior^28,43^.
Cultivar	Cultivar grown.
CO2_Treatment	Denotes whether a sample was grown at ambient [CO_2_] (‘aCO2’) or elevated [CO_2_] (‘eCO2’).
N_percent	Nitrogen concentration of sample, in percent of dry mass.
P_percent	Phosphorus concentration of sample, in percent of dry mass.
K_percent	Potassium concentration of sample, in percent of dry mass.
Ca_percent	Calcium concentration of sample, in percent of dry mass.
Mg_percent	Magnesium concentration of sample, in percent of dry mass.
Zn_ppm	Zinc concentration of sample, in ppm of dry mass.
Fe_ppm	Iron concentration of sample, in ppm of dry mass.
S_ppm	Sulfur concentration of sample, in ppm of dry mass.
B_ppm	Boron concentration of sample, in ppm of dry mass.
Mn_ppm	Manganese concentration of sample, in ppm of dry mass.
Cu_ppm	Copper concentration of sample, in ppm of dry mass.
Phy_Ext1_Precip1	First of two precipitation measurements of phytic acid phosphorus from the first of up to three phytic acid extractions, in mg g^−1^.
Phy_Ext1_Precip2	Second of two precipitation measurements of phytic acid phosphorus from the first of up to three phytic acid extractions, in mg g^−1^.
Phy_Ext1_Avg	Average of the two phytic acid phosphorus measurements from the first of up to three phytic acid extractions, in mg g^−1^.
Phy_Ext2_Precip1	First of two precipitation measurements of phytic acid phosphorus from the second of up to three phytic acid extractions, in mg g^−1^.
Phy_Ext2_Precip2	Second of two precipitation measurements of phytic acid phosphorus from the second of up to three phytic acid extractions, in mg g^−1^.
Phy_Ext2_Avg	Average of the two phytic acid phosphorus measurements from the second of up to three phytic acid extractions, in mg g^−1^.
Phy_Ext3_Precip1	First of two precipitation measurements of phytic acid phosphorus from the third of up to three phytic acid extractions, in mg g^−1^.
Phy_Ext3_Precip2	Second of two precipitation measurements of phytic acid phosphorus from the third of up to three phytic acid extractions, in mg g^−1^.
Phy_Ext3_Avg	Average of the two phytic acid phosphorus measurements from the third of up to three phytic acid extractions, in mg g^−1^.
Phy_Grand_Avg	Sample phytic acid phosphorus concentration in mg g^−1^. This is the average of the results from the first two extractions if they differed by less than 15%, otherwise a third extraction was performed and the two extractions in best agreement were averaged to generate this value.

**Table 2:** Validation of phytic acid phosphorus measurements

**Species**	**Line**	**Phytic Acid P**		
		**Ferric. Precip. (mg g**^**−1**^)	**HPLC (mg g**^**−1**^)	**HL Method (mg g**^**−1**^**±s.d.)**
Barley	Harrington	1.99	2.71	2.55±0.32
Barley	*lpa*1-1-M422	1.05	1.17	1.08±0.21
Barley	*lpa*-M955	0.15	0.15	0.25±0.11
Maize	Normal-WT	2.73	3.86	3.68±0.46
Maize	*lpa*1-1	1.2	1.65	1.36±0.21
Soybean	Jack	4.21	4.95	4.68±0.25
Soybean	CX-183	1.42	1.45	1.28±0.23
Soybean	CAPPA	0.48	0.38	0.17±0.06
Standard Deviation of the Mean	0.16 (*n*=3)	0.07 (*n*=3)	0.12 (*n*=5)	
Phytic acid phosphorus in samples used as tissue controls as determined using the ‘ferric precipitation method,’ HPLC, or the HL method used in the FACE analyses in the Raboy lab. (s.d.=Standard Deviation).				
